# The Symptomatic Goitre of Tuberculosis of the Lungs

**Published:** 1874-05-01

**Authors:** 


### THE SYMPTOMATIC GOITRE OF TUBERCULOSIS OF THE LUNGS.

*Translated for* THE EXAMINER, *from Memorabilien, Vol. XVIII., No. 12.*

DR. BETZ says: "It is not uncommon to find, in the tenth to twelfth year—indeed, even earlier—an enlargement of the thyroid gland, which is connected with tuberculosis of the lungs. This struma could easily be looked upon as a forerunner of tuberculosis, if it was not to be considered as a symptom of that disease already existing. When the symptomatic goitre occurs at the beginning, or middle, of puberty, as it frequently does with girls, it could be connected more easily with this than with affection of the lungs. To remove this struma, particularly under the last-mentioned circumstances, aid is quickly sought, and the well-known iodine remedy is used. When tuberculosis clearly makes its appearance, and the goitre has diminished in size, it is to be feared, they say, that the goitre should not have been driven away, or the iodine employed for this is the cause of the consumption." Such reports have come to the notice of other physicians, as well as himself. He therefore takes little more notice of a bronchocele, where he anticipates tuberculosis of the lungs; and he thinks he is able to do this sooner, as this form of goitre does not increase to any great size. The enlargement of the thyroid gland has its cause, not in any participation with the process of tuberculous infiltration, but in a more chemical one, viz.: a chronic stasis of the blood. When the apices of the lungs become tuberculous, they are not only retarded in their growth, but diminish in size. Herein, as it is well known, lies the cause of the consumptive thorax, which is formed more perfectly the earlier the tuberculosis begins, and the softer are the costal cartilages. Now, if tuberculosis of the lungs developes early, in the fifth or sixth year, the upper portion of the thoracic cavity is not sufficiently expanded; the clavicles, and the manubrium of the sternum are sunken in. This condition produces a pressure on the jugular veins, and in consequence there exists a circulation anomaly of the blood glands of the neck. This explanation seems to him to be the most probable. Usually, in such cases, the neck is covered more or less with pigment. Goitre is not a forerunner, but a symptom, of tuberculosis of the apices of the lungs, which has existed for a long time.

